# High Cholesterol-Induced Bone Loss Is Attenuated by Arctiin via an Action in Osteoclasts

**DOI:** 10.3390/nu14214483

**Published:** 2022-10-25

**Authors:** Guoen Li, Jung-Nam Park, Hyun-Jung Park, Jae-Hee Suh, Hye-Seon Choi

**Affiliations:** 1 Department of Biological Sciences (BK21 Program), University of Ulsan, Ulsan 44610, Korea; 2 Department of Pathology, Ulsan University Hospital, Ulsan 44030, Korea

**Keywords:** arctiin, bone loss, 7-ketocholesterol, osteoclast, oxidative stress

## Abstract

High cholesterol-induced bone loss is highly associated with oxidative stress, which leads to the generation of oxysterols, such as 7-ketocholesterol (7-KC). Here, we conducted in vivo and in vitro experiments to determine whether arctiin prevents high cholesterol diet-induced bone loss by decreasing oxidative stress. First, arctiin was orally administered to atherogenic diet (AD)-fed C57BL/6J male mice at a dose of 10 mg/kg for 6 weeks. Micro-computerized tomography (μCT) analysis showed that arctiin attenuated AD-induced boss loss. For our in vitro experiments, the anti-oxidant effects of arctiin were evaluated in 7-KC-stimulated osteoclasts (OCs). Arctiin decreased the number and activity of OCs and inhibited autophagy by disrupting the nuclear localization of transcription factor EB (TFEB) and downregulating the oxidized TFEB signaling pathway in OCs upon 7-KC stimulation. Furthermore, arctiin decreased the levels of reactive oxygen species (ROS) by enhancing the expression of nuclear factor erythroid 2-related factor 2 (Nrf2), catalase, and heme oxygenase 1 (HO-1), all of which affected OC differentiation. Conversely, silencing of Nrf2 or HO-1/catalase attenuated the effects of arctiin on OCs. Collectively, our findings suggested that arctiin attenuates 7-KC-induced osteoclastogenesis by increasing the expression of ROS scavenging genes in the Nrf2/HO-1/catalase signaling pathway, thereby decreasing OC autophagy. Moreover, arctiin inhibits the oxidation and nuclear localization of TFEB, thus protecting mice from AD-induced bone loss. Our findings thus demonstrate the therapeutic potential of arctiin for the prevention of cholesterol-induced bone loss.

## 1. Introduction

Emerging evidence has suggested a positive correlation between high cholesterol levels and decreased bone density [1,2,3]. In postmenopausal women, high low-density lipoprotein levels have been negatively associated with bone mass [4]. In contrast, statin treatment, which is commonly prescribed to reduce serum cholesterol levels, increases bone mineral density (BMD) [5]. When two strains of mice were exposed to a high-fat diet, atherosclerosis-prone mice exhibited lower bone density than an atherosclerosis-resistant strain [6], suggesting that both diseases are caused by common cholesterol metabolites. The pathophysiology of atherosclerosis has been linked to an excessive accumulation of oxidized lipids and lipoproteins in large arteries. Oxysterols are oxidized metabolites that are generated through enzymatic or non-enzymatic cholesterol degradation processes in differentiated macrophages upon oxidative stress [7,8]. A direct radical attack on cholesterol by reactive oxygen species (ROS) generates 7α/ß-hydroxycholesterol and 7-ketocholesterol (7-KC) [9], which are reported to be highly linked to the pathophysiology of atherosclerosis [10,11]. Our previous studies demonstrated that a high cholesterol diet induces bone loss [12] and increases the levels of 7-KC in bone [13]. In turn, this enhances osteoclastogenesis by stimulating autophagy, which is also partly due to increased ROS levels [13].

Arctiin, an arctigenin glycoside, is a bioactive ingredient of *Arctium lappa* L. that has long been used as a medicinal herb in East Asia [14]. Specifically, arctiin-containing plants have been widely used in traditional oriental medicine as effective treatments against rheumatic arthritis, inflammatory disease, and infection [14]. In mice, arctiin reduces obesity caused by high-fat diets by decreasing adipogenesis [15]. Moreover, oral administration of arctiin reduces neuroinflammation in mice by inhibiting nuclear factor-κB (NF-κB) activation, thereby exerting an antidepressant effect [16]. Arctiin also inhibits the activation of phosphoinositide 3-kinase and NF-κB in mice with lipopolysaccharide (LPS)-induced acute lung injury [17]. After oral injection, arctiin undergoes metabolic pathways via detachment of glucose residues, demethylation, and dihydroxylation in intestinal lumen [18]. Arctigenin, a non-glycosylated form of arctiin, is known for its potent anti-inflammatory and antiseptic properties [14]. Additionally, other metabolites of arctiin also possess antiviral (particularly anti-hepatitis B) and anticarcinogenic properties [18]. Arctiin was also reported to protect mice against ovariectomy-induced bone loss and inhibited osteoclastogenesis by decreasing receptor activator of nuclear factor-κB (RANKL) signaling in vitro [19].

Based on the aforementioned findings, we hypothesized that arctiin may prevent high cholesterol diet-induced bone loss by decreasing oxidative stress. Therefore, we investigated the effects of arctiin on bone mass in atherogenic diet-fed mice in vivo and its molecular mechanisms in osteoclasts (OCs) upon 7-KC stimulation in vitro.

## 2. Materials and Methods

### 2.1. Reagents

Arctiin was obtained from Chengdu Herb Purify Co., Ltd. (Chengdu, China). Macrophage-colony stimulating factor (M-CSF) and receptor activator of nuclear factor-κB ligand (RANKL) were purchased from R&D Systems, Inc. (Minneapolis, MN, USA). Opti-MEM and M-MLV reverse transcriptase were provided by Gibco (Grand Island, NY, USA) and Promega (Madison, WI, USA), respectively. 7-KC, a leukocyte acid phosphatase (TRAP) kit, N-Acetyl-L-cysteine (NAC), MTT (3-(4,5-dimethylthiazol-2-yl)-2,5-diphenyltetrazolium bromide), diphenyleneiodonium chloride (DPI), and toluidine blue were provided by Sigma Chemical (St. Louis, MO, USA). SYBR Green was purchased from Qiagen (Hilden, Germany). The OxiSelect™ Hydrogen Peroxide/Peroxidase Assay Kit (Colorimetric) was purchased from Cell Biolabs Inc. (San Diego, CA, USA). The RatLaps EIA kit was obtained from Immunodiagnostic Systems Inc. (Fountain Hills, AZ, USA). The TRIzol reagent, lipofectamine RNAiMAX transfection reagent, and N-(Biotinoyl)-N’-(iodoacetyl) ethylenediamine (BIAM) were acquired from Invitrogen (Carlsbad, CA, USA). An alkaline phosphatase (ALP) colorimetric kinetic assay and an osteocalcin EIA kit were purchased from BioAssay Systems (Hayward, CA, USA) and Biomedical Technologies Inc. (Stoughton, MA, USA), respectively. The SYBR Green Real-Time PCR Master Mixes, NE-PER nuclear and cytoplasmic extraction reagents, 2′,7′-dichlorofluorescein diacetate (H_2_DCFDA), and HRP-conjugated secondary antibodies were acquired from Thermo Fisher Scientific (Waltham, MA, USA). Antibodies against lamin B1 (#13435) and microtubule-associated proteins 1A/1B light chain 3B (LC3B; #2775) were provided by Cell Signaling (Danvers, MA, USA). Antibodies against β-actin (A5441) and transcription factor EB (TFEB; A303-673A) were purchased from Sigma Chemical and Bethyl Laboratories Inc (Montgomery, TX, USA), respectively. The scrambled siRNA (scRNA; sc-37007), nuclear factor erythroid 2-related factor 2 (Nrf2) small interfering RNA (siRNA) (siNrf2; sc-37049), heme oxygenase 1 (HO-1) siRNA (siHO-1; sc-35555), catalase siRNA (sicatalase; sc-45331), peroxiredoxin (PRDX) 1 siRNA (siPRDX1; sc-36178), PRDX4 siRNA (siPRDX4; sc-40836), and horseradish peroxidase (HRP)-streptavidin were provided by Santa Cruz Biotechnology (Santa Cruz, CA, USA).

### 2.2. Study Design and Animals

The C57BL/6J male mice, ten-weeks-old, were obtained from The Jackson Laboratory (Hana, Busan, Korea) and the mice were fed with either a chow (a normal diet, ND) or atherogenic diet (AD) for 6 weeks. The mouse AD was provided by Research Diets, Inc. (New Brunswick, NJ, USA). For X-ray radiogram, the mice were randomly divided into five groups: ND + phosphate-buffered saline (PBS) (*n* = 4), AD + PBS (*n* = 4), and AD + arctiin (5 mg/kg, *n* = 4; 10 mg/kg, *n* = 4; 30mg/kg, *n* = 4). Arctiin was dissolved in dimethyl sulfoxide (DMSO) and adjusted to a 0.5% DMSO concentration by diluting it with PBS; the mice were orally treated with different concentrations of arctiin (or just PBS as a vehicle control) every day for 6 weeks. For micro-computed tomography (μCT) and serum extraction, the animals were randomly divided into the following four groups: ND + PBS (*n* = 5), ND + arctiin (*n* = 5), AD + PBS (*n* = 5), and AD + arctiin (*n* = 5). The mice were orally treated with 10 mg/kg of arctiin or PBS every day for 6 weeks. All mice were kept in a specific pathogen-free environment. All standards employed herein were approved by the UOUACUC (HSC-21-010). In addition, all animal care and operations were performed in accordance with protocols and guidelines accepted by the Animal Care and Use Committee at the University of Ulsan (UOUACUC). After 6 weeks, in order to acquire serum, the mice were anesthetized, and a heparinized capillary tube was used to retro-orbitally collect a blood sample. Then, the mice were euthanized with CO_2_ and the femurs were harvested for X-ray radiogram or μCT analysis. For radiographic analysis, mouse femora were cleaned and stored in 10% formalin for a week. Femur radiographs were taken by soft X ray (model CMB-2; SOFTEX) according to the manufacturers’ instructions. Relative intensities of the distal femurs from X-ray radiograms were measured by using the ImageJ (version 1.37) software. To visualize long bone architecture, femurs were analyzed by scanning with a high-resolution μCT imaging system in a SkyScan 1072 System (SkyScan, Kontich, Belgium) set to a 6.9 μm effective detector pixel size and a 77–255 mg/cc threshold. Trabecular bone was analyzed in a 1.7 mm region located 0.4 mm below the distal femur growth plate. Three-dimensional analyses were performed with the CT volume software (ver. 1.11; SkyScan) using a total of 75–125 tomographic slices. To examine in vivo TRAP-positive OCs, mouse femora were excised, and decalcified in EDTA. Representative histologic sections of the distal femoral metaphysis of mice fed with AD or ND in the presence or absence of arctiin were stained for TRAP and hematoxylin and eosin (H&E) to identify OCs (original magnification: ×200 and ×400) and growth plates (original magnification: ×40 and ×100), respectively. Serum C-telopeptide fragments of collagen type 1 (CTX-1) were measured as an in vivo marker of bone resorption using the RatLaps enzyme immunoassay (EIA). Serum ALP and osteocalcin were measured with a colorimetric kinetic assay and an osteocalcin EIA kit, respectively. Serum H_2_O_2_ was measured with the OxiSelect™ Hydrogen Peroxide/Peroxidase Assay Kit (Colorimetric). All experiments were carried out according to the manufacturers’ instructions.

### 2.3. OC Formation

Bone marrow cells were obtained from six-week-old C57BL/6J male mice as previously described [20]. To obtain bone marrow cells, the femur and tibia were aseptically excised; a 1 mL syringe was inserted at one end of the bone and the marrow cavity was flushed with PBS or α-MEM. Next, the harvested cells were incubated in complete medium (10% FBS and 100 U/mL of penicillin/streptomycin in α-MEM) containing M-CSF (30 ng/mL) for 16–24 h in an incubator at 37 °C and 5% CO_2_. The next day, floating cells were collected and transferred to plates in complete medium containing M-CSF (30 ng/mL) for 48 h. Then, M-CSF (30 ng/mL) and RANKL (40 ng/mL) were added to the BMMs, and the cells were fixed in 10% formalin for 10 min after incubation for the times indicated in each figure legend. The cells were then stained for tartrate-resistant alkaline phosphatase (TRAP) as described in a previous study [20]. The area and maximum diameter of the formed OCs were measured using the ImageJ (version 1.37) software and the fusion index was presented as the average number of nuclei per TRAP-positive MNC [21]. The number of OCs was determined by counting TRAP-positive multinucleated (three or more nuclei) cells (MNCs) per well using an eyepiece graticule.

### 2.4. Cell Viability Assays

The effect of arctiin on cell viability was determined by MTT assay. Briefly, the BMMs were treated with different concentrations of arctiin (10, 15, and 20 μM) in the presence of M-CSF (30 ng/mL), RANKL (40 ng/mL) and 7-KC (7 μM) until mature osteoclasts were formed. Then, the cells were treated with 0.5 mg/mL MTT (3-(4,5-dimethylthiazol-2,5-diphenyltetrazoliu bromide)) at 37 °C for 30 min. After removing the medium, DMSO (80 μL) was added to the cells and the absorbance was measured at 540 nm using a SpectraMax^®^ iD3 multi-mode microplate reader (Molecular Devices, San Jose, CA, USA).

### 2.5. Bone Resorption

To assess the effect of arctiin on the function of OCs, we further implanted mature OCs on dentine slices for 4 to 5 days as previously described [22]. In short, BMMs were incubated with M-CSF (30 ng/mL) and RANKL (40 ng/mL) until mature OCs were formed. The mature OCs were then harvested and seeded on dentine slices before being incubated with RANKL and M-CSF in the presence or absence of 7-KC (7 μM) or arctiin (15 μM) for 4–5 days. The cells were then fixed with 10% formalin and stained for TRAP. Afterward, the cells were removed via ultrasonication in 1 M NH_4_OH and the dentine slices were stained with 1% (*w*/*v*) toluidine blue in 0.5% sodium borate to visualize the resorption pits. The resorption pit area and TRAP-positive cells were measured with the ImageJ (version 1.37) software.

### 2.6. RNA Extraction and Quantitative Real-Time PCR (qPCR)

To synthesize single-stranded cDNA, total RNA was extracted and reverse-transcribed using M-MLV reverse transcriptase and random primers. QPCR was performed using SYBR Green (Qiagen, Hilden, Germany) with the appropriate primers. The specific primers used are shown in Supplementary Table S1. The expression of the housekeeping gene 18S ribosomal RNA (RPS) was used to normalize the relative amount of mRNA expression. The 2^−∆∆Ct^ method was used to calculate the copy numbers relative to RPS expression.

### 2.7. Western Blot Analysis

To evaluate the localization of TFEB and the expression of LC3B, the cell proteins were extracted by using NE-PER nuclear and cytoplasmic extraction reagents and radioimmune precipitation lysis buffer, respectively. SDS-PAGE was used to separate the protein, which was then transferred to a nitrocellulose membrane. After that, the membranes were blocked for 1 h at room temperature with 5% skim milk in Tris-buffered saline containing 0.1% Tween 20 (1X TBS-T). Next, the specific primary antibodies against LC3B (1:1000), β-actin (1:10,000), TFEB (1:1000), and lamin B1 (1:1000) were incubated at 4 °C for overnight with shaking, then the membranes were washed with 1X TBS-T and incubated with secondary antibodies for 1 h. Finally, the membranes were developed using chemiluminescent substrates after being washed three times with 1X TBS-T. The intensity of the bands was determined using ImageJ (version 1.37) software.

### 2.8. Flow Cytometry Analysis of Acidic Vesicular Organelles Stained with Acridine Orange

To investigate the effect of arctiin on autolysosomes, flow cytometry was utilized to analyze acidic vesicular organelles (AVOs; i.e., autolysosomes) after staining with acridine orange (AO), as previously described [23]. AO is a weakly fluorescent base that accumulates in acidic environments and emits a strong red fluorescent signal. The cells were stained with AO, the nucleolus and cytoplasm show dim red and bright green, respectively, whereas AVOs appear brilliant red. The degree of acidity and volume of AVOs are proportional to the intensity of red fluorescence. In order to assess AVOs, BMMs were incubated with M-CSF (30 ng/mL), RANKL (40 ng/mL), and 7-KC (7 μM) in the presence of arctiin (15 μM) until mature osteoclasts developed, and complete medium was refreshed every two days. In addition, the cells were starved for 4 h without FBS as a positive control and for 4 h with bafilomycin A1 (BafA1) as a negative control. The cells were then stained with AO (1μg/mL) at 37 °C for 20 min, washed twice with PBS, and determined using a flow cytometer.

### 2.9. Detection of Intracellular Reactive Oxygen Species (ROS)

The fluorescent probe 2′,7′-dichlorofluorescein diacetate (H_2_DCFDA) was used to determine intracellular ROS. In brief, BMMs were cultured with M-CSF (30 ng/mL), RANKL (40 ng/mL), and 7-KC (7 μM) in the presence of different concentrations of arctiin (10, 15, 20 μM) for 48 h. The cells were then washed twice with PBS and treated with fresh α-MEM containing 10 μM H2DCFDA and incubated at 37 °C in the dark for 10–15 min. Intracellular ROS were detected by using a FACSCalibur flow cytometer (Becton Dickinson, Franklin Lakes, NJ, USA).

### 2.10. Determination of Oxidized TFEB via Carboxymethylation

BMMs were treated with 7-KC (7 μM) ± arctiin (15 μM) in the presence of M-CSF (30 ng/mL) and RANKL (40 ng/mL) for 51 h. The cells were then harvested and flash-frozen in liquid nitrogen. Next, the frozen cells were transferred to a lysis buffer containing 100 μM BIAM and the solution was deoxygenated for 20 min by bubbling nitrogen gas at a low flow rate. BIAM is extensively employed as a sulfhydryl modifying agent that selectively detects the reduced form of cysteine [24]. The extracted proteins were added to 2 μg of TFEB-specific antibody for immunoprecipitation. HRP-conjugated streptavidin was utilized to identify BIAM-tagged immunocomplexes, which were then developed using a chemiluminescence kit.

### 2.11. SiRNA Transfection

BMMs were treated with M-CSF (30 ng/mL), RANKL (40 ng/mL), and 7-KC (7 μM) in the presence or absence of arctiin (15 μM) for 3 h and then transfected with siRNA against Nrf2 or HO-1 and catalase or with scRNA as a negative control using the lipofectamine RNAiMAX transfection reagent according to the manufacturer’s instructions. After transfection, the cells were cultured with M-CSF (30 ng/mL), RANKL (40 ng/mL), and 7-KC (7 μM) with or without arctiin (15 μM) for the times indicated in the figure legends.

### 2.12. Statistical Analysis

All data were presented as means ± standard deviation (SD). The differences between the samples and the corresponding controls were assessed by using Student’s *t*-test. One-way ANOVA was used to evaluate group differences followed by Bonferroni post-hoc tests. A statistically significant difference was considered as a *p*-value < 0.05.

## 3. Results

### 3.1. Arctiin Protects Mice from AD-Induced Bone Loss

**Figure 1:**
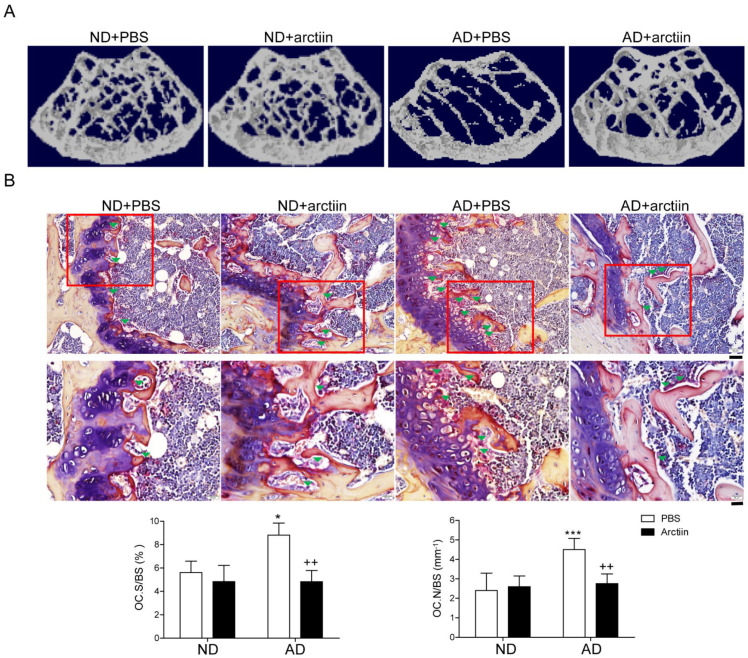
Arctiin protects mice from atherogenic diet-induced bone loss. (**A**) Representative μCT images of distal mouse femora area (1.7 mm) located 0.4 mm below the distal femur growth plate in mice fed with ND + PBS (*n* = 5), ND + arctiin (10 mg/kg/d, *n* = 5), AD + PBS (*n* = 5), or AD + arctiin (10 mg/kg/d, *n* = 5) for 6 weeks. (**B**) Representative histologic sections of the distal femoral metaphysis of each group were stained for TRAP to calculate the OC.N/BS (OC number over total bone surface) and OC.S/BS (OC surface over total bone surface) ratios (original magnification ×200 (top) and ×400 (bottom)). Scale bar: ×200 (50 μm), ×400 (20 μm) in representative photos. * *p* < 0.05; *** *p* < 0.001 compared with the corresponding ND group. *^++^ p* < 0.01 compared with the corresponding AD group. Differences between groups were analyzed via two-way ANOVA followed by Bonferroni post hoc tests. Similar results were obtained in three independent sets of experiments and μCT analysis was performed with a representative set.

To investigate whether arctiin prevented cholesterol-rich AD-induced bone loss, mice fed with either a high cholesterol diet (AD; 1.25% cholesterol and 0.5% Na cholate) or an isocaloric low-cholesterol control diet (normal diet, ND) for 6 weeks were orally treated with arctiin or vehicle (PBS). A 10 mg/kg dose of arctiin treatment exhibited maximum protection compared to doses of 5 mg/kg and 30 mg/kg, as demonstrated by X-ray radiogram analyses (Supplementary Figure S1A). Moreover, no significant difference of body weight was observed between four groups at 16 weeks of age. μCT scans of femurs from mice fed with AD showed a significant bone loss with decreased bone mineral density (BMD), bone volume (BV/TV), trabecular thickness (Tb. Th), and trabecular number (Tb. N.) and increased trabecular separation (Tb. Sp.) compared to mice fed with ND, whereas injection of arctiin attenuated bone loss in mice fed with AD. In particular, treatment with arctiin definitely increased the BMD, BV/TV, Tb. Th, and Tb. N. while decreasing Tb. Sp. when compared to mice fed with AD only (Figure 1A and Table 1). However, arctiin exhibited no significant effect on mice fed with the ND (Figure 1A and Table 1). According to our in vivo TRAP staining analyses, AD-fed mice exhibited higher OC.S/BS (the ratio of OC surface to total bone surface area) and OC.N/BS (the ratio of OC number to total bone surface area) compared to ND-fed mice (Figure 1B). In contrast, arctiin administration reduced the OC.S/BS and OC.N/BS ratios that were enhanced in the AD-fed mice but not in mice fed with the ND (Figure 1B). No significant change among 4 groups was observed in growth plates by H&E staining (Supplementary Figure S1B). In agreement with these findings, oral injection of arctiin attenuated the expression of CTX-1 (an in vivo bone resorption marker) that was enhanced in mice fed with AD, but no significant change was observed in mice fed with the ND. Furthermore, the in vivo bone formation markers ALP and osteocalcin that were increased in mice fed with AD compared to those fed with ND did not change significantly in the arctiin-treated mice (Table 1). In contrast, the augmented serum levels of ROS in AD-fed mice were significantly reduced upon arctiin administration compared to the vehicle (PBS) treatment (Table 1).

### 3.2. Arctiin Decreases the Number and Activity of OCs That Are Augmented by 7-KC

**Figure 2:**
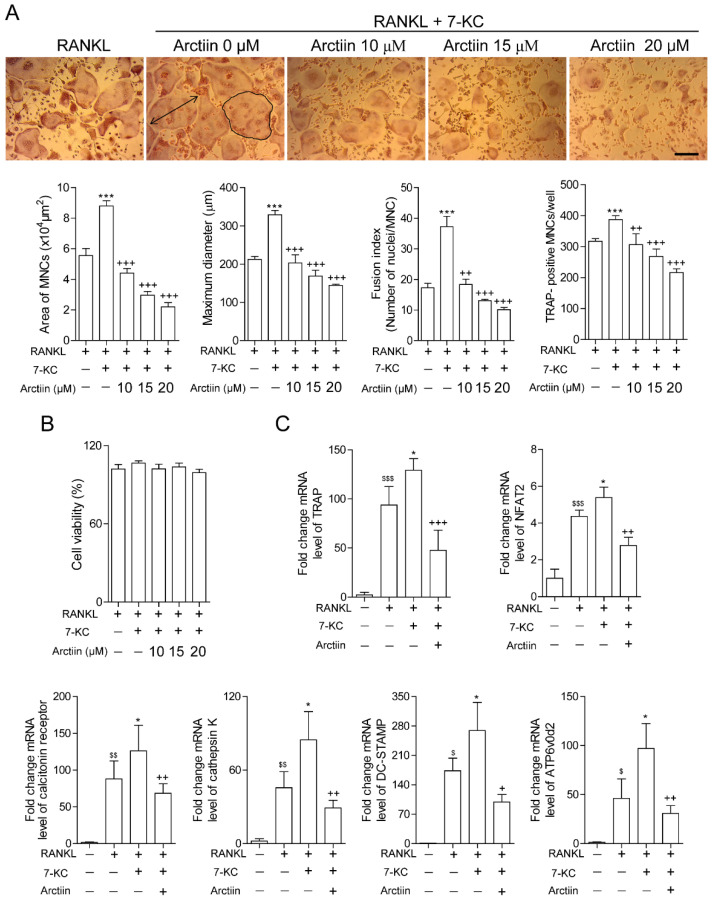

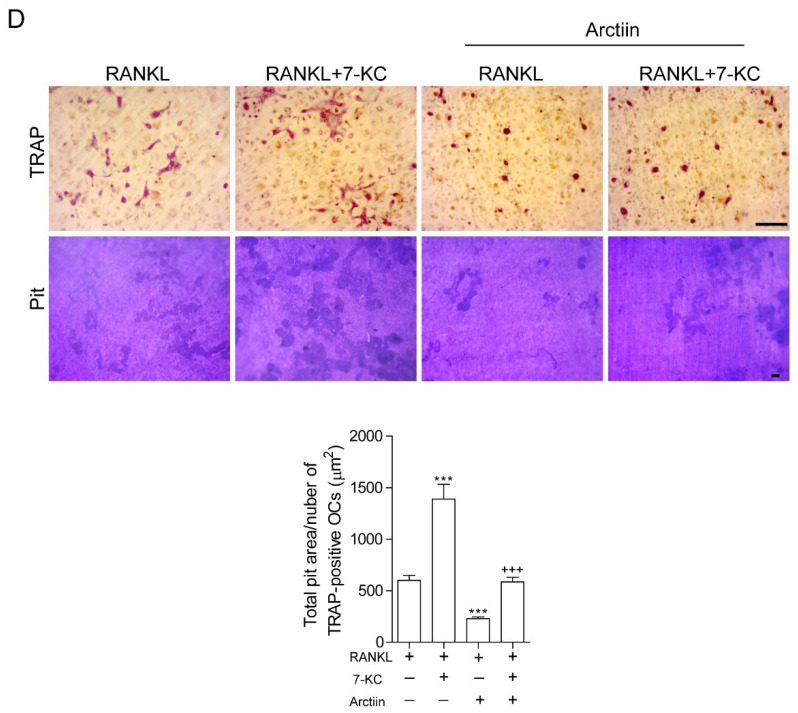
Arctiin decreases the number and activity of osteoclasts that are augmented by 7-KC. (**A**) BMMs were incubated with M-CSF (30 ng/mL) and RANKL (40 ng/mL) in the presence or absence of 7-KC (7 μM) or arctiin (10, 15, 20 μM) for 65 h (*n* = 3). After TRAP staining, the area (bold line), maximum diameter (double arrow), fusion index, and number of OCs were measured. Representative photos are shown (scale bar: 200 μm). (**B**) Cell viability was measured via the MTT assay (*n* = 5). (**C**) RNA from cells stimulated with M-CSF (30 ng/mL) and RANKL (40 ng/mL) in the presence or absence of 7-KC (7 μM) or arctiin (15 μM) for 65 h was analyzed by qPCR (*n* = 4). The expression level before RANKL treatment was set to 1. (**D**) Mature OCs were further incubated on whole dentine slices with M-CSF (30 ng/mL) and RANKL (40 ng/mL) in the presence or absence of 7-KC (7 μM) or arctiin (15 μM) for 4–5 d (*n* = 3). Representative photos of TRAP-positive OCs (scale bar: 200 μm) and resorption pits (scale bar: 50 μm) are shown. The total pit area/number of TRAP-positive OCs was calculated. ^$^ *p* < 0.05; ^$$^ *p* < 0.01; ^$$$^ *p* < 0.001 compared with BMMs. * *p* < 0.05; *** *p* < 0.001 compared with RANKL-treated cells. ^+^ *p* < 0.05; ^++^ *p* < 0.01; ^+++^ *p* < 0.001 compared with RANKL and 7-KC-treated cells. Similar results were obtained in three independent sets of experiments.

Our previous data suggested that increased dietary cholesterol decreased BMD [12] with enhance the levels of 7-KC in bone [13]. Given that arctiin prevented high cholesterol-induced bone loss in mice, which coincided with a decrease in the number and surface of OCs, we hypothesized that arctiin may act by lessening the effects of 7-KC in OCs. To evaluate the role of arctiin in osteoclastogenesis, we next sought to characterize the dose-dependent effects of arctiin on osteoclast differentiation and cell cytotoxicity. As shown in Figure 2A, arctiin significantly and dose-dependently decreased OC surface area, maximum diameter, and fusion index, as well as the number of OCs that were enhanced upon 7-KC stimulation. Arctiin treatment had no visible detrimental effects on cell viability at the range of concentration used in the current study (Figure 2B). In addition, we also found that arctiin decreased the expression of OC-specific genes at the mRNA level, including TRAP, NFAT2, calcitonin receptor, cathepsin K, DC-STAMP, and ATP6v0d2, which were upregulated in 7-KC-treated cells (Figure 2C). Next, we examined whether arctiin affected in vitro 7-KC-enhanced bone resorption. As we expected, arctiin treatment dramatically reduced the total pit area/number of OCs when compared to cells stimulated without arctiin (Figure 2D).

### 3.3. Arctiin Decreases Autophagy by Disrupting 7-KC-Stimulated TFEB Nuclear Localization

**Figure 3:**
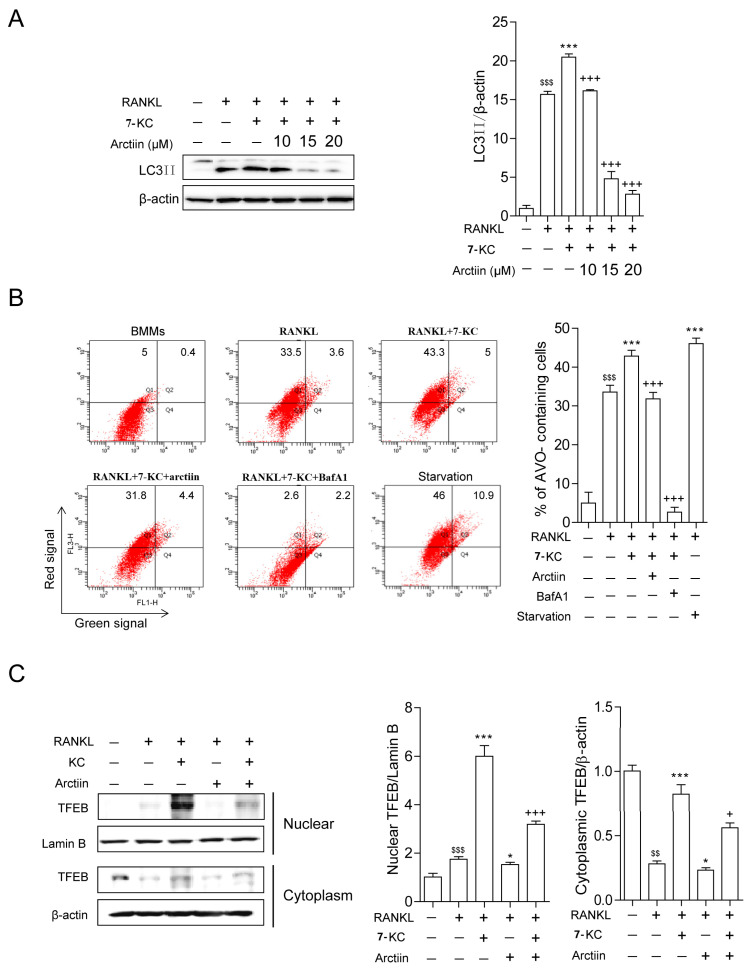
Arctiin decreases autophagy by disrupting 7-KC-stimulated TFEB nuclear localization. (**A**) BMMs were incubated with M-CSF (30 ng/mL) and RANKL (40 ng/mL) in the presence or absence of 7-KC (7 μM) or arctiin (10, 15, 20 μM) for 65 h. The levels of LC3II were analyzed in cell lysates. BafA1 (25 nM) was added 4 h before harvesting to stimulate the accumulation of LC3II. The expression of LC3II was normalized to that of β-actin. (**B**) 7-KC-induced AVO formation with arctiin (15 μM) was analyzed by flow cytometry. The values represent the percentage of AVO-containing cells in representative experiments. (**C**) Whole-cell extracts were harvested from cultured cells, fractionated into cytoplasmic and nuclear parts, and subjected to Western blot analysis with anti-TFEB antibody. Antibodies for β-actin and lamin B1 were used for the normalization of cytoplasmic and nuclear extracts, respectively ((**A**–**C**); *n* = 3). ^$$^ *p* *<* 0.01; ^$$$^ *p* *<* 0.001 compared with BMMs. * *p <* 0.05; *** *p <* 0.001 compared with RANKL-treated cells. *^+^ p <* 0.05; *^+++^ p <* 0.001 compared with RANKL and 7-KC-treated cells. Similar results were obtained in three independent sets of experiments.

Our previous results revealed that 7-KC enhanced autophagy by facilitating TFEB nuclear localization in OCs [13]. Therefore, we now sought to determine whether arctiin attenuated this effect. As shown in Figure 3A, arctiin dose-dependently reduced the expression of lipidated form of LC3 (LC3II) that were enhanced by 7-KC stimulation. LC3II has been reported to be a reliable marker of autophagic flux. Particularly, BafA1 treatment prevents the fusion of autophagosomes with lysosomes, resulting in LC3II accumulation. To investigate whether arctiin affects the formation of autolysosomes (i.e., the result of autophagosome and lysosome fusion), autolysosome formation was analyzed by characterizing the fractions of AVO (including autolysosomes)-containing cells using flow cytometry coupled with the pH-sensitive AO fluorescent dye. Arctiin treatment significantly decreased the percentage of AVO-containing cells, whereas 7-KC markedly increased it (Figure 3B). Starvation and BafA1 were used as positive and negative controls, respectively. Next, we examined whether arctiin lowered TFEB nuclear localization, which was enhanced upon 7-KC stimulation. As shown in Figure 3C, 7-KC increased the nuclear localization of TFEB compared to RANKL alone, whereas addition of arctiin significantly down-regulated it.

### 3.4. Arctiin Decreases Cytosolic ROS That Is Increased upon 7-KC Stimulation, Leading to Disrupted TFEB Nuclear Localization via Decreased Oxidation

**Figure 4:**
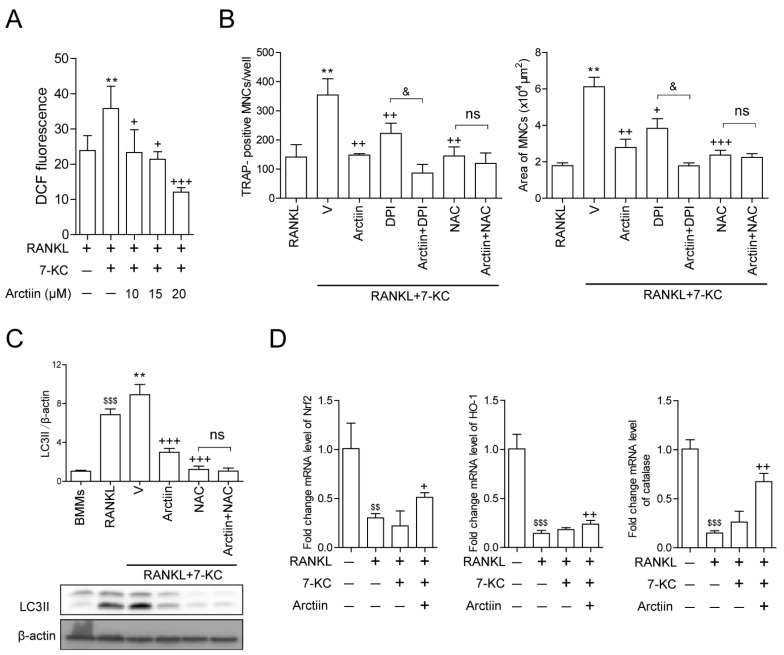

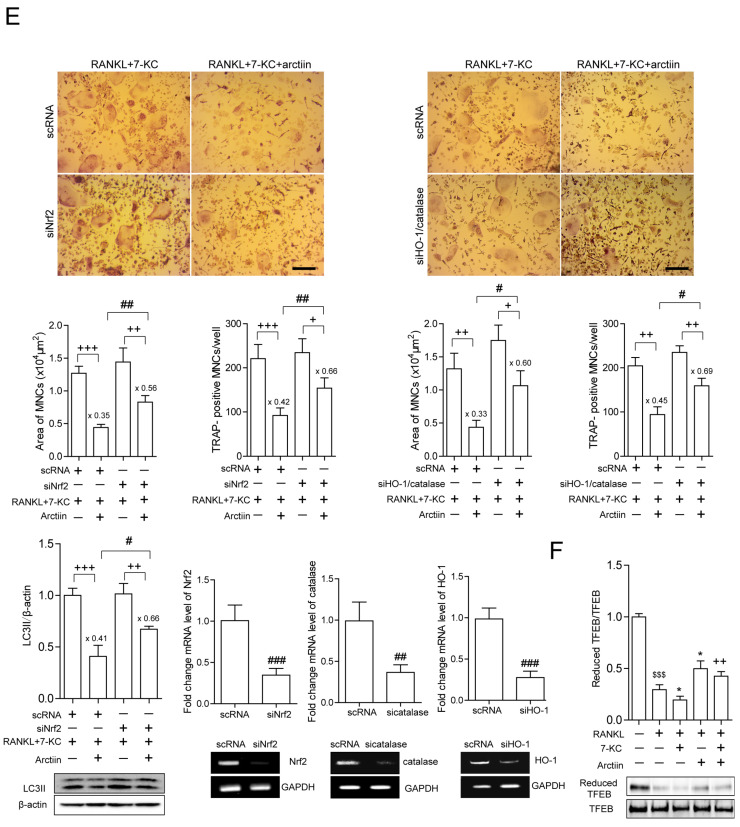
Arctiin decreases cytosolic ROS that is increased upon 7-KC stimulation, leading to an increase in the reduced form of TFEB. (**A**) Cytosolic ROS levels were determined by flow cytometry using H_2_DCFDA after 48 h with arctiin (10, 15, 20 μM) (*n* ≥ 3). (**B**,**C**) BMMs were incubated with M-CSF (30 ng/mL) and RANKL (40 ng/mL) in the presence or absence of 7-KC (7 μM) or arctiin (15 μM) ± DPI (5 nM) or NAC (3 mM) for 65 h (*n* = 3). (**B**) The cells were then fixed, TRAP-positive MNCs and the OC area were analyzed. (**C**) The levels of LC3II were analyzed in the cell lysates. BafA1 (25 nM) was added 4 h before harvesting to stimulate the accumulation of LC3II. The expression of LC3II was normalized to that of β-actin. (**D**) RNA from cells stimulated with M-CSF (30 ng/mL), RANKL (40 ng/mL), and 7-KC (7 μM) in the presence of arctiin (15 μM) for 48 h was analyzed by qPCR. The expression level before RANKL treatment was set to 1 (*n* ≥ 3). (**E**) BMMs were transfected with scRNA, siNrf2, or combination of catalase and HO-1 and further incubated with M-CSF (30 ng/mL), RANKL (40 ng/mL), and 7-KC (7 μM) in the presence or absence of arctiin (15 μM) (*n* ≥ 3). The cells were fixed after 65 h, TRAP-positive MNCs and the OC area were analyzed. Representative photos are shown (scale bar: 200 μm). The levels of LC3II were analyzed in cell lysates after 65 h. BafA1 (25 nM) was added 4 h before harvesting to stimulate the accumulation of LC3II. The down-regulation of Nrf2, catalase, and HO-1 by siRNA was confirmed by reverse transcription (RT)-PCR and qPCR. (**F**) BMMs were incubated with M-CSF (30 ng/mL) and RANKL (40 ng/mL) in the presence or absence of 7-KC (7 μM) or arctiin (15 μM) (*n* = 3) for 51 h. The cell lysates were labeled with N-(biotinoyl)-N’-(iodoacetyl) ethylenediamine, and TFEB was immunoprecipitated (IP) from each sample. HRP-streptavidin immunoblotting was performed to quantify the levels of the reduced form of TFEB. ^$$^ *p* < 0.01; ^$$$^ *p* < 0.001 compared with BMMs. * *p* < 0.05; ** *p* < 0.01 compared with RANKL-treated cells. ^+^ *p* < 0.05; ^++^ *p* < 0.01; ^+++^ *p* < 0.001 compared with RANKL and 7-KC-treated cells. ^&^ *p* < 0.05 compared with the corresponding control. ^#^ *p* < 0.05; ^##^ *p* < 0.01; ^###^ *p* < 0.001 compared with scRNA-treated cells. Ns, no significance. Similar results were obtained in three independent sets of experiments.

We next sought to investigate the molecular mechanisms through which arctiin affected OCs. In our previous study, we demonstrated that the stimulatory effects of 7-KC on OCs were due to its capacity to increase ROS levels [13]. Therefore, we hypothesized that arctiin decreases autophagy by modulating ROS levels. As expected, arctiin decreased cytosolic ROS after 48 h of 7-KC and RANKL exposure in a dose-dependent manner (Figure 4A). To identify a potential target of arctiin to decrease cytosolic ROS levels, cells treated with 7-KC and RANKL were co-treated with DPI (an NADPH oxidase inhibitor) or NAC (a ROS scavenger) in the presence or absence of arctiin. Arctiin and DPI decreased the surface area and number of OCs in an additive manner, whereas when arctiin and NAC were combined, no additional reduction was observed compared with NAC alone (Figure 4B). The levels of LC3II exhibited a similar pattern in OCs (Figure 4C). Based on these findings, we next sought to identify which antioxidants were up-regulated by arctiin to reduce ROS levels. We found that arctiin significantly up-regulated the expression levels of the antioxidants Nrf2, HO-1, catalase, PRDX1, and PRDX4 but not those of PRDX2, PRDX3, PRDX5, PRDX6, GPX1, SOD1, SOD2, and TRX1 (Figure 4D and Supplementary Figure S2). When Nrf1 only or a combination of catalase and HO-1 was down regulated, the negative effect of arctiin on OC area and number was partially decreased, whereas inhibition the expression of Nrf2 or catalase and HO-1 trended to increase the number and area of osteoclasts compared to the scRNA-treated control upon 7-KC stimulation, but there was no statistical difference (Figure 4E). Silencing of Nrf2 also attenuated the negative effect of arctiin on LC3II level (Figure 4E). However, knock-down of PRDX1 or PRDX4 had no significant effects on the activity of arctiin on OCs (Supplementary Figure S3). Next, carboxymethylation analysis was performed to assess whether decreased ROS by arctiin affected the level of oxidized TFEB. As explicitly observed in Figure 4F, RANKL decreased the reduced form of TFEB and the addition of 7-KC further decreased it, whereas arctiin treatment enhanced the levels of the reduced form of TFEB. A similar pattern was found with NAC in our previous study [13].

## 4. Discussion

Our findings demonstrated that arctiin, an arctigenin glycoside purified from *Arctium lappa* [14], protected mice from cholesterol-induced bone loss. Our previous studies showed that cholesterol-rich AD induced bone loss in mice, which coincided with an increased number and surface of OCs [12]. Our in vivo study demonstrated that the number and surface of OCs in bone and the serum level of the bone resorption marker CTX-1 were significantly reduced when arctiin was orally administered compared to the vehicle (PBS)-treated group in AD-fed mice, whereas in vivo bone formation markers (serum ALP and osteocalcin) exhibited no significant changes, suggesting that arctiin decreases cholesterol-induced bone loss in mice mainly by affecting OC activity. However, we cannot exclude that the effect of arctiin could be partly due to inhibition of RANKL signaling as demonstrated [19]. In addition, H&E staining showed that no significant change was found in growth plates of high cholesterol-fed groups compared to normal cholesterol-fed groups, although dysregulated cholesterol has been reported to induce cartilage degradation via chondrocytes during osteoarthritis development [25,26]. However, the morphology of the growth plates has not been further studied in detail, including immunohistochemistry, such as MMP13 [27]. In our in vitro experiments, arctiin decreased the number, area, maximum diameter, and fusion index of 7-KC-stimulated OCs. Augmented bone resorption stimulated by 7-KC was also significantly reduced by arctiin in dentine slices. These results suggested that arctiin affects both the activity and differentiation of OCs. Furthermore, arctiin decreased both 7-KC-enhanced LC3II levels and fractions of AVO-containing cells, indicating that arctiin reduced 7-KC-stimulated autophagy formation and subsequent fusion with lysosomes. These findings indicated that arctiin lowered the number and activity of OCs by decreasing autophagy in OCs that were stimulated by 7-KC treatment, which ultimately had a protective effect against cholesterol-induced bone loss.

Our previous study demonstrated that AD-induced bone loss was accompanied by oxidative stress as well as increased 7-KC levels in bone [13], suggesting that 7-KC, an in vivo autooxidation product of cholesterol, may play a role in bone loss. The level of 7-KC affected not only the differentiation but also the activity of OCs in vitro, in addition to promoting autophagy by up-regulating ROS [13]. 7-KC increased the level of oxidized TFEB, which facilitated nuclear localization of TFEB, resulting in augmented autophagy in OCs [13]. TFEB has been reported to act as a transcription factor that promotes the expression of genes involved in the autophagy-lysosome system [28,29], and nuclear localization of TFEB can be regulated by phosphorylation [30] as well as oxidation [13,31]. Limit of our study could be due to not to determine the 7-KC levels in bone after arctiin treatment in vivo. However, our study demonstrated that arctiin decreased nuclear localization of TFEB, as well as the level of oxidized TFEB enhanced by 7-KC. When DPI (an inhibitor of NADPH oxidase) was added to the cells to block the production of ROS, arctiin and DPI decreased additively the OC area and number that were increased by 7-KC, indicating that the effect of arctiin was independent of ROS generation induced by 7-KC. Removal of ROS by NAC (an antioxidant) completely abolished the effect of arctiin, suggesting that the effects of arctiin were largely attributable to its capacity to enhance antioxidant activity. PRDXs (PRDX1 to PRDX6) are a family of antioxidant proteins that maintain redox balance by removing hydrogen peroxide [32]. When macrophages were submitted to oxidative stress, PRDX1 was highly expressed in the cytosol [33]. Moreover, PRDX1 deficiency not only induced oxidative stress and impaired macrophage lipophagic flux [34] but also decreased autophagy by attenuating NF-KB activation in cancer cells [35]. Upon Nrf2 activation, Nrf2 translocates to the nucleus, facilitating the expression of antioxidants with ARE binding sites in their promoters including HO-1, catalase, SOD, and GPX [36], acting as an upstream promoter for HO-1 and catalase. As expected, arctiin significantly increased the expression of HO-1, catalase, and Nrf2, as well as PRDX1 and PRDX4, compared with 7-KC only. Therefore, gene silencing experiments were performed to confirm the regulatory role of arctiin on the activity of HO-1, catalase, Nrf2, PRDX1, and PRDX4 in OCs. Knock-down of Nrf2 or HO-1 and catalase attenuated the effect of arctiin on OCs, whereas down-regulation of PRDX1 or PRDX4 had no appreciable effects. These results suggested that Nrf2 was responsible for the activity of arctiin on OCs by acting as an upstream molecule of HO-1 and catalase. In agreement with our findings, the protective effects of arctiin against OC differentiation upon RANKL stimulation [19] and against inflammation in H9N2 virus-infected cells [37] have been previously linked to the activation of Nrf2 and its target genes. Furthermore, arctiin also can protect against triptolide-induced hepatotoxicity by acting as an upstream activator to regulate the Nrf2 signaling pathway [38].

## 5. Conclusions

**Figure 5:**
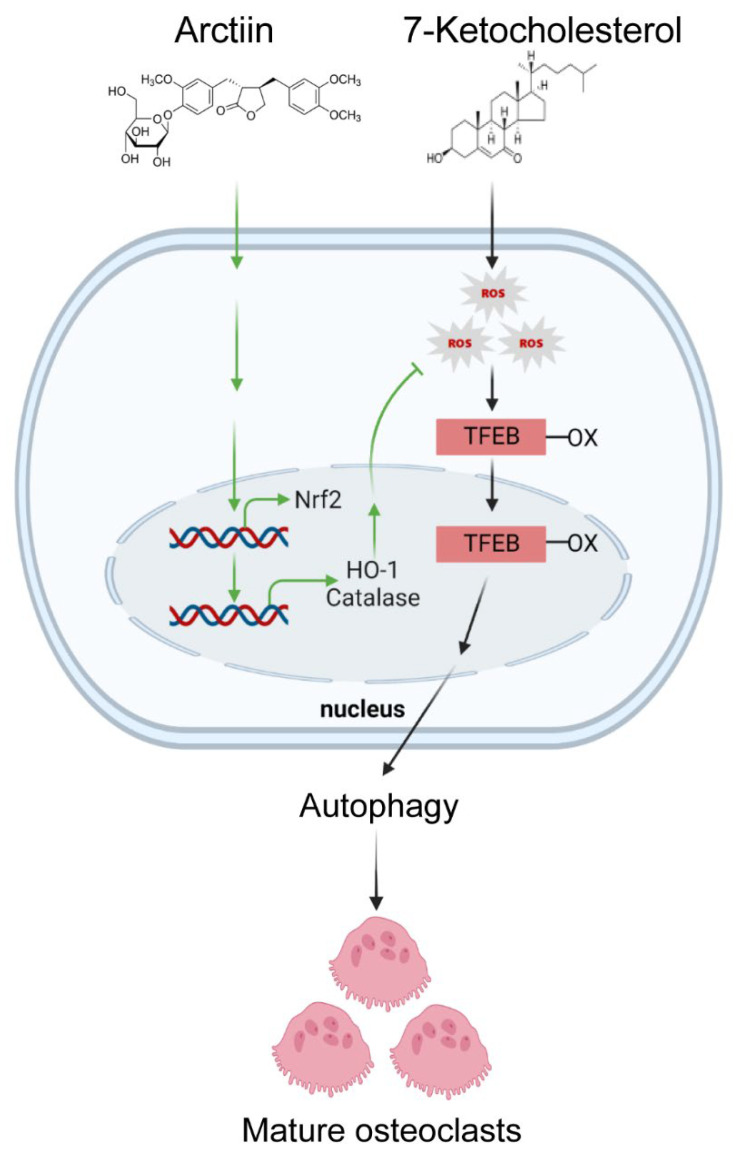
Arctiin attenuates the effects of 7-KC by decreasing the oxidized TFEB signaling pathway, thereby disturbing TFEB nuclear localization. Moreover, arctiin increases the expression of Nrf2, catalase, and HO-1, resulting in decreased ROS and subsequently impairing the activity of 7-KC in OCs, thus highlighting the therapeutic potential of arctiin to prevent cholesterol-induced bone loss.

Collectively, our findings, at least partly, demonstrated that arctiin up-regulates the expression of Nrf2 and its target genes including HO-1 and catalase, thereby decreasing ROS levels in OCs and inhibiting cholesterol-induced bone loss. Furthermore, arctiin abrogates autophagy by disrupting 7-KC-stimulated TFEB nuclear localization via decreasing oxidation in OCs (Figure 5). Therefore, our findings imply that arctiin could be used as a potential therapeutic agent to prevent high cholesterol-induced bone loss.

## Figures and Tables

**Table 1:** Trabecular microarchitecture and biochemical markers of ND and AD-fed mice with or without arctiin treatment.

Parameter	ND		AD	
	*PBS*	*Arctiin*	*PBS*	*Arctiin*
BMD [mg/cm^3^]	270.7 ± 16.23	266 ± 4.00	192.8 ± 7.11 ***	232.3 ± 9.10 ^++^
BV/TV [%]	23.00 ± 1.44	23.03 ± 0.42	16.43 ± 0.51 ***	20.25 ± 1.01 ^++^
Tb.Th [μm]	75.58 ± 2.47	73.19 ± 2.05	66.10 ± 1.04 **	74.92 ± 1.34 ^+++^
Tb.N [mm^−1^]	3.04 ± 0.12	3.15 ± 0.04	2.441 ± 0.06 ***	2.71 ± 0.09 ^+^
Tb.Sp [μm]	253.1 ± 10.50	250.2 ± 3.71	388.2 ± 17.45 **	326.4 ± 18.30 ^+^
ALP [U/L]	30.01 ± 0.36	30.43 ± 3.59	53.75 ± 3.25 ***	55.22 ± 1.89
OCN [ng/mL]	18.60 ± 0.86	20.95 ± 2.76	27.37 ± 2.16 *	23.33 ± 2.48
CTX-1 [ng/mL]	17.25 ± 1.94	16.03 ± 2.70	26.81 ± 1.30 **	19.27 ± 1.23 ^+^
H_2_O_2_ [μM]	60.86 ± 2.41	59.08 ± 1.15	69.29 ± 0.69 **	61.72 ± 1.83 ^++^

ND + PBS (*n* = 5); ND + arctiin (dissolved in DMSO, 10mg/kg) (*n* = 5); AD + PBS (*n* = 5); AD + arctiin (dissolved in DMSO, 10mg/kg) (*n* = 5). Differences between groups were analyzed by two-way ANOVA, followed by Bonferroni post hoc tests. * *p* < 0.05; ** *p <* 0.01; *** *p <* 0.001 compared with ND group. *^+^ p <* 0.05; *^++^ p <* 0.01; *^+++^ p <* 0.001 compared with AD group.

## Data Availability

All data and original images are contained within the article.

## Supplementary Materials

The following supporting information can be downloaded at: https://www.mdpi.com/article/10.3390/nu14214483/s1, Supplementary Table S1: Primer sequences used for real-time RT-PCR. Supplementary Figure S1: Arctiin protects mice from atherogenic diet-induced bone loss. Supplementary Figure S2: Arctiin increases the expression of PRDX1 and PRDX4 but does not affect the expression of PRDX2, PRDX3, PRDX5, PRDX6, GPX-1, SOD1, SOD2, and TRX1. Supplementary Figure S3: Inhibition of PRDX1 or PRDX4 expression does not affect the activity of arctiin on OC differentiation. Supplementary Figure S4: The entire uncropped images of the original Western blots and reverse transcription polymerase chain reaction.
