# Supplementary material for: High Cholesterol-Induced Bone Loss Is Attenuated by Arctiin via an Action in Osteoclasts
Source: Nutrients. 2022 Oct 25;14(21):4483. doi: 10.3390/nu14214483 (PMC9657919; doi:10.3390/nu14214483)
Supplement: Supplementary file 1 [file nutrients-14-04483-s001.zip › nutrients-1938476-supplementary.pdf]

Supplementary Table S1: Primer sequences used for real-time RT-PCR.

Genes	Forward 5'-3'	Reverse 5'-3'
**ATP6v0d2**	**TTC AGT TGC TAT CCA GGA CTC GGA**	GCA TGT CAT GTA GGT GAG AAA TGT GCT CA
Cathepsin K	GGG CCA GGA TGA AAG TTG TA	CAC TGC TCT CTT CAG GGC TT
Calcitonin receptor	AGT TGC CCT CTT ATG AAG GAG AAG	GGA GTG TCG TCC CAG CAC AT
DC-STAMP	TCC TCC ATG AAC AAA CAG TTC CAA	AGA CGT GGT TTA GGA ATG CAG CTC
NFAT2	AAT AAC ATG CGA GCC ATC ATC	TCA CCC TGG TGT TCT TCC TC
TRAP	GAC CAC CTT GGC AAT GTC TCT G	TGG CTG AGG AAG TCA TCT GAG TTG
Nrf2	CTC GCT GGA AAA AGA AGT G	CCG TCC AGG AGT TCA GAG G
HO-1	GCC GAG AAT GCT GAG TTC ATG	TGG TAC AAG GAA GCC ATC ACC
Catalase	CCA GCG ACC AGA TGA AGC AG	CCA CTC TCT CAG GAA TCC GC-3
TRX1	TTT CCA TCT GGT TCT GCT GAG A	TGG AAG AAG GGC TTG ATC ATT T
GPX1	CGC TCT TTA CCT TCC TGC GGA A	AGT TCC AGG CAA TGT CGT TGC G
SOD1	GGT GAA CCA GTT GTG TTG TCA GG	ATG AGG TCC TGC ACT GGT ACA G
SOD2	TAA CGC GCA GAT CAT GCA GCT G	AGG CTG AAG AGC GAC CTG AGT T
PRDX1	TGC CAG ATG GAC AAT TCA AA	CAG CTG GAC ACA CTT CAC CA
PRDX2	AAC GCG CAA ATC GGA AAG T	AGT CCT CAG CAT GGT CGC TAA
PRDX3	GGC CAC ATG AAC ATC ACA CTG T	CAA ACT GGA ACG CCT TTA CCA
PRDX4	TCC TGT TGC GGA CCG AAT	GAT CTT GGC TTT GCT TAG ATG CA
PRDX5	GAA AGA AGC AGG TTG GGA GTG T	CCC AGG GAC TCC AAA CAA AA
PRDX6	TTG ATG ATA AGG GCA GGG AC	CTA CCA TCA CGC TCT CTC CC
RPS	ATC AGA GAG TTG ACC GCA GTT G	AAT GAA CCG AAG CAC ACC ATA G

Note: ATP6v0d2, (H+ATPaseV0 subunit d2 [V-ATPase-d2]; DC-STAMP, dendritic cell–specific transmembrane protein; NFAT2, nuclear factor of activated T cells 1; TRAP, tartrate-resistant acid phosphatase; Nrf2, nuclear factor erythroid 2-related factor 2; HO-1, heme oxygenase-1; TRX1, thioredoxin 1; GPX1, glutathione peroxidase 1; SOD, superoxide dismutase; PRDX, peroxiredoxin; RPS, 18S ribosomal RNA.

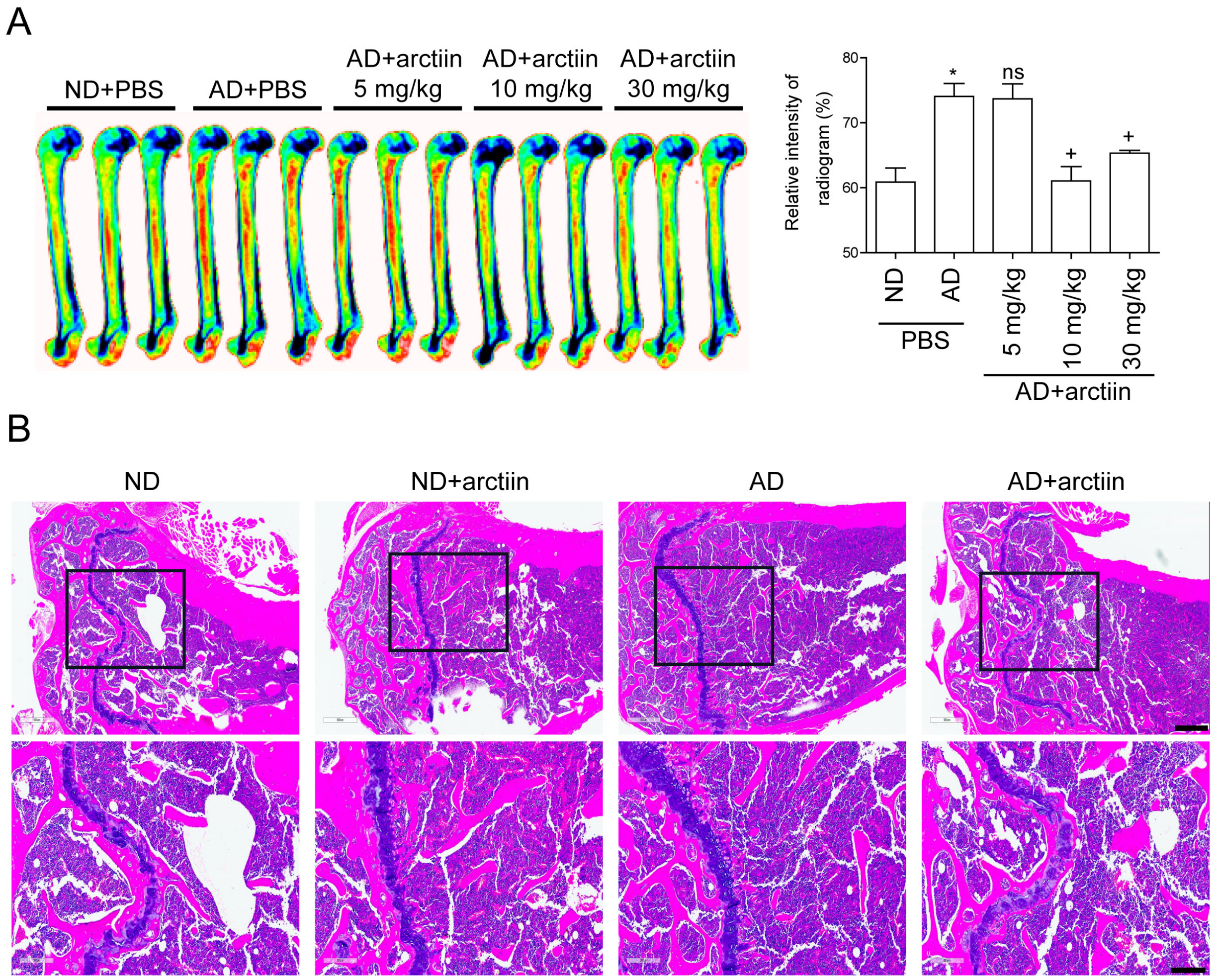

Supplementary Figure S1. Arctiin protects mice from atherogenic diet-induced bone loss. (**A**) Representative images and relative intensities of the distal femurs from X-ray radiograms in mice fed with a normal diet (ND) + PBS (n = 4), an atherogenic diet (AD) + PBS (n = 4), or AD + arctiin (5 mg/kg/d, n = 4; 10 mg/kg/d, n = 4; 30 mg/kg/d, n = 4) for six weeks were measured using the ImageJ program. Radiogram was taken with each of three representative mice. (**B**) Representative H&E staining images of distal femoral in mice fed with ND + PBS (n = 5), ND + arctiin (10 mg/kg/d, n = 5), AD + PBS (n = 5), or AD + arctiin (10 mg/kg/d, n = 5) for six weeks. Scale bar: ×40 (top, 500 μm), ×100 (bottom, 200 μm) in representative photos. * $p < 0.05$ compared with the corresponding ND group. $^{+}$ $p < 0.05$; $^{++}$ $p < 0.01$ compared with the corresponding AD group. Ns, no significance.

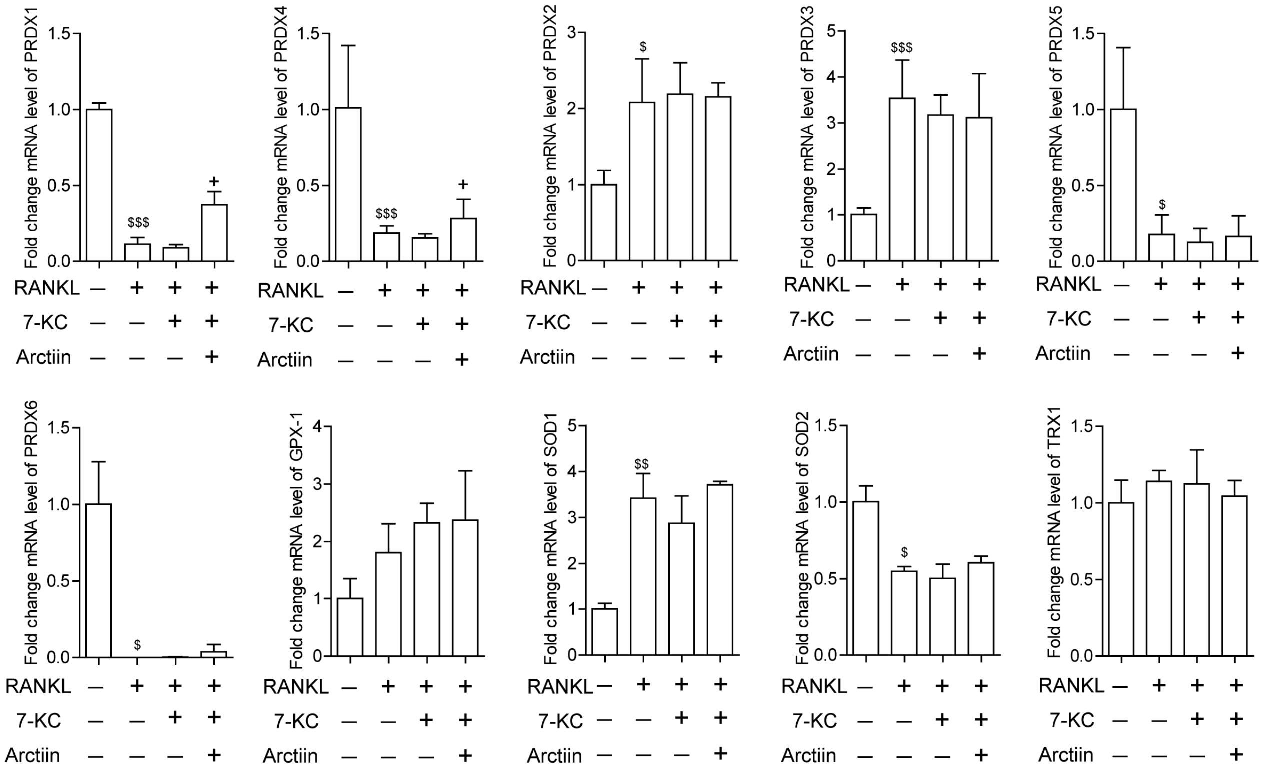

Supplementary Figure S2. Arctiin increases the expression of PRDX1 and PRDX4 but does not affect the expression of PRDX2, PRDX3, PRDX5, PRDX6, GPX-1, SOD1, SOD2, and TRX1. RNA from cells stimulated with M-CSF (30 ng/ml), RANKL (40 ng/ml), and 7-KC (7 μM) in the presence of arctiin (15 μM) for 48 h was analyzed by qPCR. The expression level before RANKL treatment was set to 1. $^{\$}$ $p < 0.05$; $^{\$\$}$ $p < 0.01$; $^{\$\$\$}$ $p < 0.001$ compared with BMMs. $^{+}$ $p < 0.05$ compared with RANKL and 7-KC-treated cells. Similar results were obtained in three independent sets of experiments.

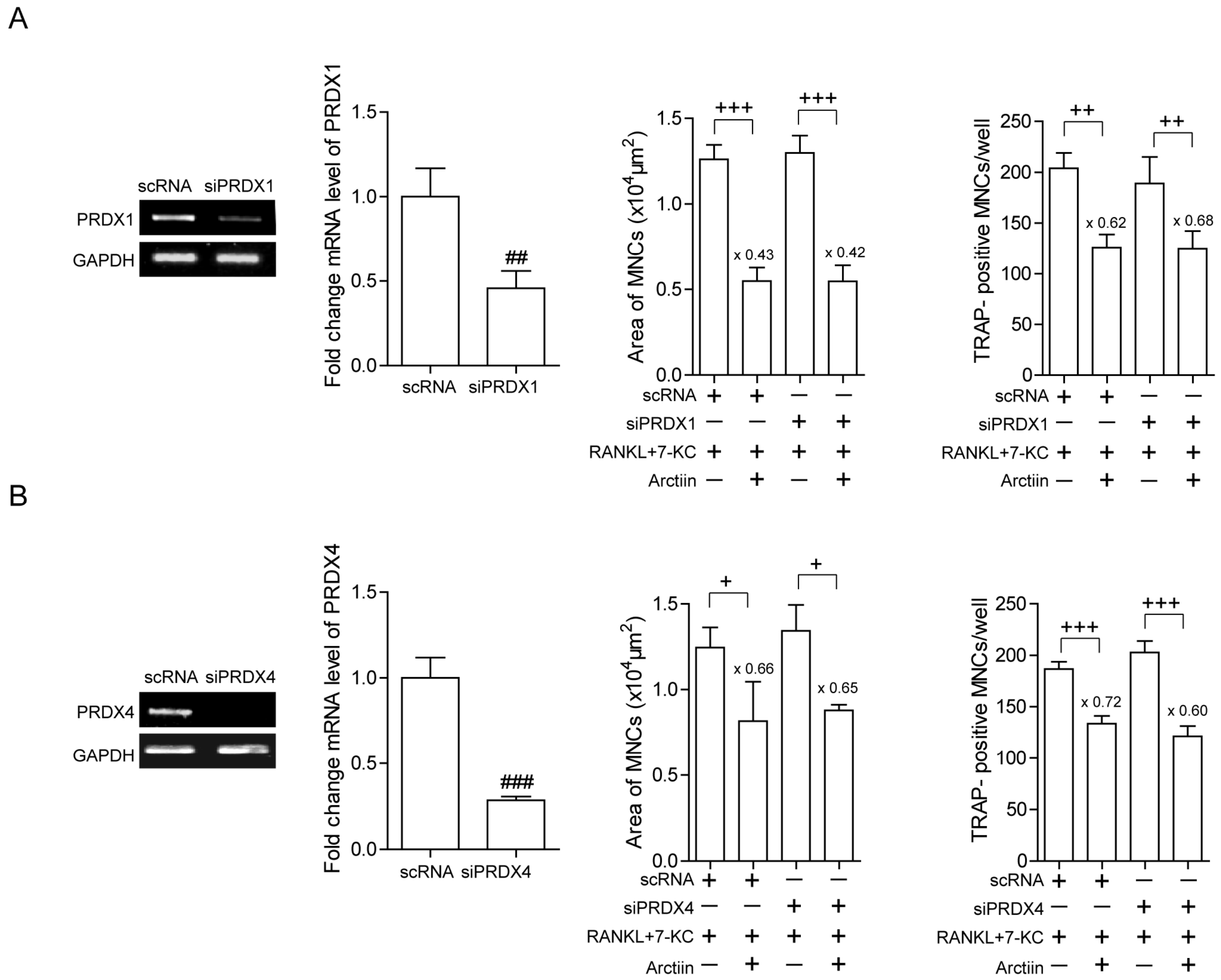

Supplementary Figure S3. Inhibition of PRDX1 or PRDX4 expression does not affect the activity of arctiin on OC differentiation. (**A**,**B**) BMMs were transfected with scRNA, siPRDX1, or PRDX4 and further incubated with M-CSF (30 ng/ml), RANKL (40 ng/ml), and 7-KC (7 μM) in the presence or absence of arctiin (15 μM). The down-regulation of PRDX1 and PRDX4 by siRNA was confirmed by reverse transcription (RT)-PCR and qPCR. The cells were fixed after 65 h, and more than 100 TRAP-positive MNCs in each culture were randomly selected. TRAP-positive MNCs and the OC area were analyzed. $^{+}p < 0.05$; $^{++}p < 0.01$; $^{+++}p < 0.001$ compared with RANKL and 7-KC-treated cells. $^{\#\#}p < 0.01$; $^{\#\#\#}p < 0.001$ compared with the scRNA-treated cells. Similar results were obtained in three independent sets of experiments.

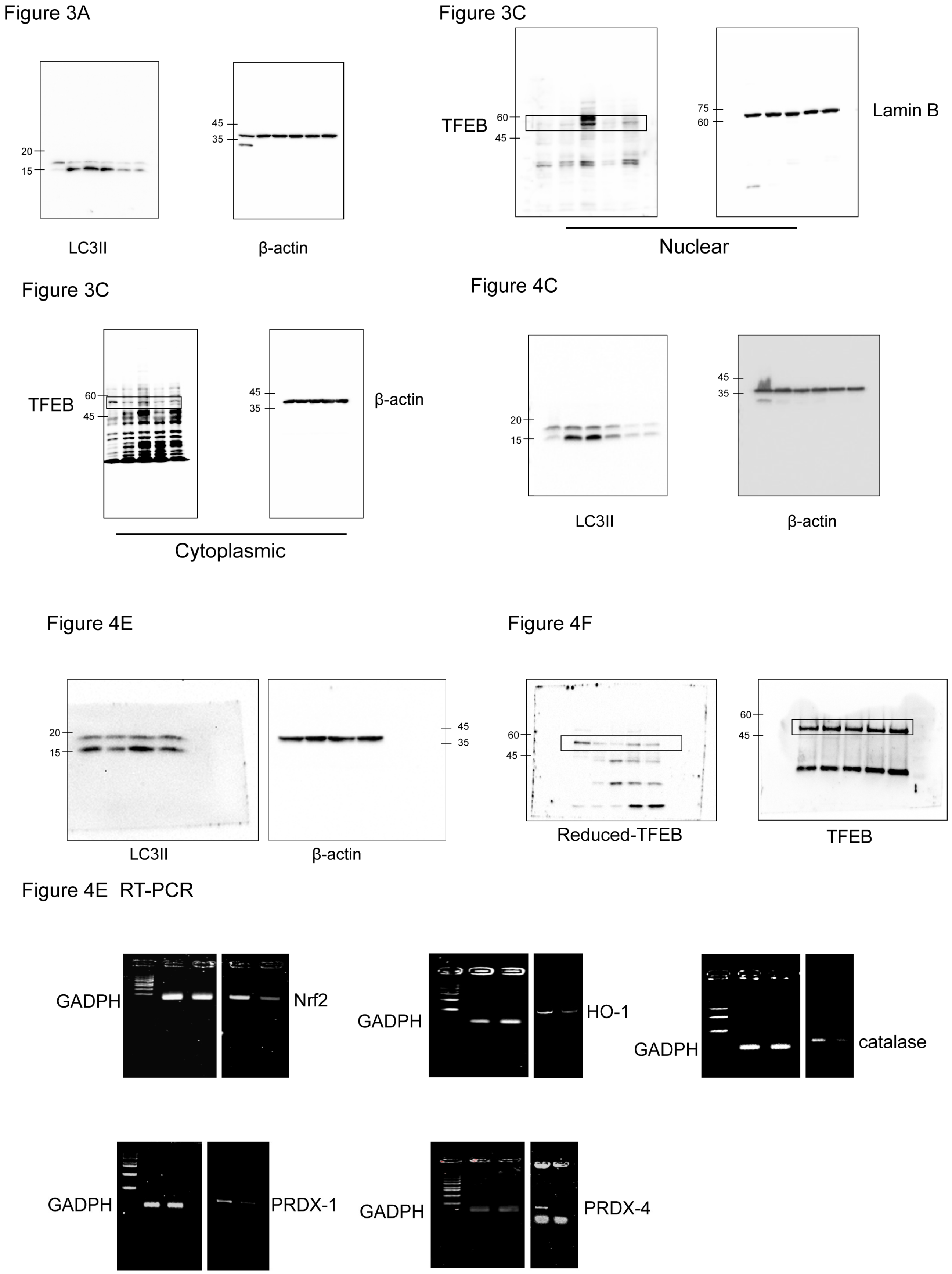

Supplementary Figure S4. The entire uncropped images of the original Western blots and reverse transcription polymerase chain reaction.
